# The step-to-step transition mode: A potential indicator of first-fall risk in elderly adults?

**DOI:** 10.1371/journal.pone.0220791

**Published:** 2019-08-02

**Authors:** Guillaume M. Meurisse, Guillaume J. Bastien, Bénédicte Schepens

**Affiliations:** Laboratory of Physiology and Biomechanics of Locomotion, Institute of Neuroscience (IoNS), Université catholique de Louvain (UCL), Louvain-la-Neuve, Belgium; Toronto Rehabilitation Institute - UHN, CANADA

## Introduction

More than 25% of elderly adults fall each year [1] and these falls are often associated with serious injuries and hospitalizations [2]. Knowing that the percentage of worldwide population over 60 year-old will continue to rise, the prevention of falls is a major issue for our society.

The most commonly fall prediction factor, as well as the best single predictor, is the history of falling [3, 4]. However, despite its high predictive power, it is irrelevant to identify the first-fall risk [5]. Nevertheless, this detection is essential: the first-fall increases the risk of falling by three [5] and can result in a fear of further falling leading to self-restriction of physical and social activities [6]. Other usual fall indicators are screening tools based on an evaluation of daily movements (e.g. gait, changing position, turning reaching, standing, etc). But according to Gates et al. [7], there is no strong evidence that those screening tools are valid to identify fallers. For example, the large number of variants and cut-offs of the Tinetti Test [8] complicates its validation for falls prediction [9]. Moreover, these screening tools are based on subjective analysis requiring the presence of a trained, self-reliant expert [10] and are limited by ceiling effect [11].

In contrast, measuring instruments during standing tasks allow an objective and sensitive evaluation of the subject’s postural stability. For example, the measure of the center of pressure excursion of the subject using a force platform can objectify the postural control. Using this measure, Melzer et al. [12] have shown that balance testing in narrow stance is able to distinguish elderly adults who experience recurrent falls from non-falling elderly adults.

Beside balance tests, gait analysis is now recognized as a powerful tool to identify markers of early pathology and maximize healthy ageing. Gait may seem like a simple task because of its automatic and rhythmic nature but it is actually a complex task requiring the integration of inputs from multiple neurological structures [13]. Ageing and pathology can impact one or more of these neurological levels leading to impairments of the gait performance in a subtle way [14]. As for balance, quantitative gait measurements are useful to support the clinician in the detection of slight changes.

The evaluation of gait is also interesting for fall prediction since a large proportion of elderly adults falls occurs during walking. More precisely, most of falls occur by tripping on an external obstacle during the single support phase or by an incorrect body weight transfer from one leg to the other when elderly adults are simply walking forward [15, 16]. A first definition of the body weight transfer, based on the vertical ground reaction forces, is the double contact phase (DC) when both feet are on the ground, i.e. from the contact of the front foot (FC) to the toe-off of the (contralateral) back foot (TO). A second definition of the body weight transfer, based on the variation of the center of mass of the body (CoM) velocity, is the step-to-step transition [17] delimited by the time where the CoM reaches its minimal vertical velocity before FC and its maximal vertical velocity after TO [18]. This second definition corresponds to the redirection of the downward trajectory of the CoM in the late single-stance phase to an upward trajectory after DC. This step-to-step transition seems to be a key phase of walking, as it requires balance control to achieve the transfer of the body weight from on leg to the other. According to Chastan et al. [19], the step-to-step transition presents two different modes: an active and a passive mode. The transition is considered as active when the minimal vertical velocity of the CoM occurs before FC thanks to the activation of the ankle plantar flexors [19, 20]. This active push-off of the back leg allows an anticipated and balanced transition: the redirection of the CoM is initiated before FC and the contributions of the front and the back legs are equal. This active mode, used by healthy young adults, eases the impact of the front leg, reduces stress on the leg joint and minimizes the work necessary to lift the CoM during DC [17, 20, 21]. A greater proportion of the active transition occurs before and after DC as the walking speed increases [22]. Alternatively, the transition is considered as passive when minimal vertical velocity of the CoM occurs at or after FC; in that case the push-off of the back leg is not sufficient to initiate the transition which is initiated during DC and the force generated by the front leg is larger that the force generated by the back leg. This passive mode has been observed in young children under 5-6 years [23], in elderly adults [24] and in patients suffering from postural instability [20, 25].

During the transition, elderly adults exhibit a smaller vertical push-off done by the back leg, a greater impact on the front leg and are unable to accelerate the CoM forward and upward simultaneously [24]. Nevertheless when observing in detail the data of Meurisse et al. [24], the great intragroup variability in elderly adults during the transition could indicate different transition modes and different first-fall risks in the aged population.

The aim of this study is to use the transition mode to identify two groups in the aged population. We hypothesize that such population is made of subjects with an active transition or with a passive transition, the latter being associated with a lower force generated by the back and a largest force generated by front legs during the transfer of the body weight. We anticipate that elderly adults with passive mode will have a poorer result on static balance parameters. These results could lead to the use of the transition mode as a potential indicator of first-fall risk during walking.

## Materials and methods

### Subjects and experimental procedures

Fifteen young (age: 21 to 29 years old, weight: 68.6±14.6 kg; height: 1.73±0.09 m, BMI: 22.6±3.4 kg.m^-2^) and thirty-six elderly (age: 67 to 86 years old, weight: 69.7±13.6 kg; height: 1.70±0.09 m, BMI: 23.9±3.1 kg.m^-2^) adults were enrolled in the study. There was no significant difference between the two groups in terms of body mass, height and BMI. The inclusion criteria were: ability to walk a kilometer, no locomotor system injury complaints, no previous history of neurological disorders and a body mass index between 15 and 35 kg/m^2^. Before the experiments, the purpose and the nature of the study were explained to the participants. The experiments were performed according to the Declaration of Helsinki and were approved by the local ethics committee (“Commission d’éthique Biomédicale Hospitalo-Facultaire de l’Université catholique de Louvain”, 2015/18MAI/245, Belgian Registration Number: B403201524765). All subjects gave their written informed consent to participate in the study.

The participants were first asked to walk at their natural pace along a 12-meters walkway completing 3 trials in order to measure the spontaneous speed. Thereafter, they were asked to take part in the Performed Oriented Mobility Assessment proposed by Tinetti [8] and finally to walk on an instrumented treadmill at 1.11 m.s^-1^. Elderly adults were equipped with a harness during tests. All participants performed a warm up trial on the treadmill before data acquisition. Data were recorded for 15 s after 3 minutes of walking at constant speed.

### Experimental set-up and signals processing

#### The ground reaction forces

The ground reaction forces (GRF) were measured by means of four force transducers (Arsalis, Belgium) located under each corner of a modified commercial treadmill (h/p/Cosmos-Stellar, Germany, belt surface: 1.60x0.65 m). The forces were filtered with an 8^th^ order Bessel dual low pass filter with a cut-off frequency of 20 Hz. The non-linearity was <1% of full scale, and the crosstalk between vertical and horizontal GRF <1%.

The GRF signal was digitized with a 16-bit A/D convertor at a sampling rate of 500 Hz. The amplified GRF signals were processed by means of a computer with dedicated software (LABVIEW 2010, National Instruments, Austin, TX, USA). The decomposition algorithm proposed by Meurisse et al. [26] was used to determine the beginning and the end of the DC phase, the front FC time and the back foot TO time, and the vertical component of the GRF acting upon each limb during the double contact phase.

The GRFs were used to compute the velocity and the displacement of the CoM [27]. Data were normalized relative to the stride duration, 0% corresponding to the initial contact of the right foot and 100% corresponding to the next contact of the same foot.

### Temporal and kinetic parameters

#### Temporal parameters

The spontaneous speed was measured by two photocells placed at the level of the neck and separated by 5 meters in the middle of a 12-meters corridor. Step duration was calculated as the duration between two successive heel strikes and step frequency was obtained by taking the inverse of the step duration. DC was delimited by the front FC and the back foot TO for each weight transfer from one leg to the other (i.e. from the left leg to the right and from the right to the left). The step-to-step transition phase was delimited by the time where the CoM reaches its minimal vertical velocity before FC and its maximal vertical velocity after TO as defined by Franz and Kram [18]. Note that by definition the step-to-step transition is at minimum equal to DC. The time of occurrence of the minimal vertical velocity of the CoM (Vv_min_) relative to FC expressed in milliseconds was also calculated. The transition mode is defined by the time of occurrence of the Vv_min_ relative to the beginning of the double contact phase (FC). When Vv_min_ occurs before FC, the transition is ‘active’ while when Vv_min_ occurs at or after FC, the transition is ‘passive’.

#### Kinetic parameters

The force ratio between F_FRONT_/F_BACK_ was calculated as the ratio between the mean values of impact force on the front leg during the step-to-step transition (F_FRONT_) and the mean values of the vertical push-off performed by the back leg during this transition (F_BACK_) in order to illustrate the asymmetry in the force generation between the two legs [24].

#### CoM hodograph

The vertical and forward components of the CoM velocity were plotted against each other over the course of the step to form a CoM hodograph as described by Adamczyk and Kuo [22].

### Balance parameters

During the Performed Oriented Mobility Assessment, standing with eyes closed and standing with feet together were objectified via measuring instruments for seven young and nineteen elderly adults. During both tests, the center of pressure excursion of the subject was measured during 30 seconds by means of four force transducers (Arsalis, Belgium) located under each corner of a 1 m* 1 m force plate. The CoP was sampled at 1000 Hz and processed with a 8^th^ order Bessel dual low pass filter with a cut-off frequency of 10 Hz as recommended by Ruhe et al. [28].

### Statistics

The spontaneous speed of each subject was calculated as the mean of the three trials recorded. When walking on the treadmill, variables were averaged for 21 to 34 consecutive steps (i.e. alternating steps starting with either a left or a right foot contact). For all variables, the mean values were calculated for each group of subjects, as presented in the result section and figures. A one-way ANOVA with Bonferroni post-hoc (PASW Statistics 21, SPSS, IBM, Armonk, NY, USA) was performed to evaluate the effect of the group on the different parameters. The normality of the residuals was checked with a Kolmogorov-Smirnov test. Pearson’s correlations were performed to evaluate the relationships between balance parameters, step frequency, spontaneous speed, F_FRONT_/F_BACK_ ratio and the time of occurrence of Vv_min_ relative to FC. The level of significance was fixed to p ≤ 0.05.

## Results

### The step-to-step transition mode

**Fig 1:**
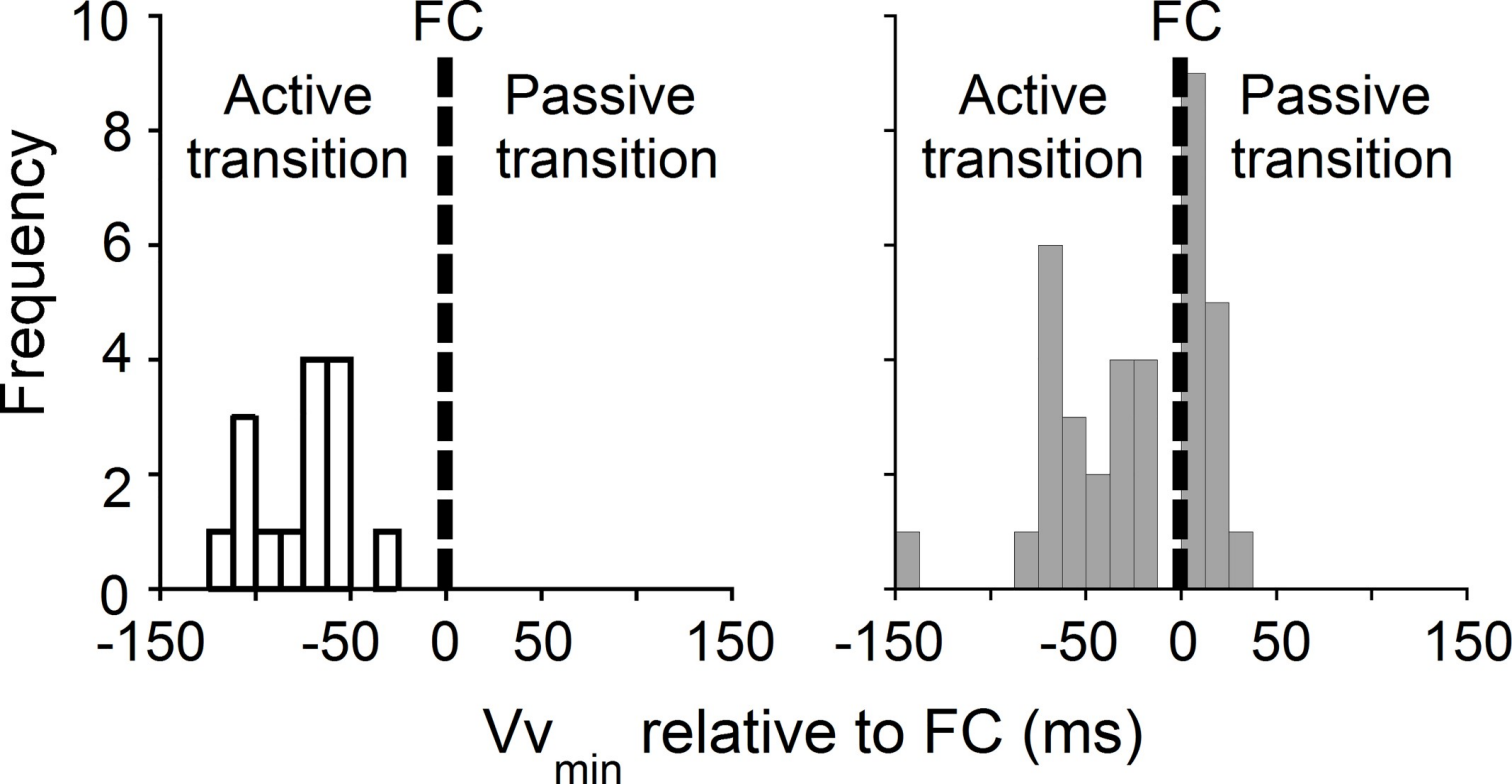
Time of occurrence distribution of Vv_min_ relative to FC of young and elderly adults. Time of occurrence distribution of the minimal vertical velocity of the CoM (Vv_min_) relative to the beginning of the double contact phase (the front foot contact, FC) expressed in milliseconds. Negative values indicate active transition; i.e. Vv_min_ occurs before FC while positive values indicate passive transition; i.e. Vv_min_ occurs after FC. Left: Distribution of young adults (white bars, n = 15). Right: Distribution of elderly adults (grey bars, n = 36: 21 with an active transition and 15 with a passive transition).

All young adults have an active transition while elderly adults could be separated in two groups: twenty-one elderly adults (age: 67 to 86 yo, weight: 67.5±13.0 kg; height: 1.68±0.09 m, BMI: 23.6±3.1 kg.m^-2^) present an active transition and fifteen elderly adults (age: 69 to 84 yo, weight: 72.9±14.4 kg; height: 1.72±0.09 m, BMI: 24.4±3.3 kg.m^-2^) present a passive transition (Fig 1). There is no significant difference between the two groups of elderly adults in terms of age, body mass, height and BMI.

### Kinetics

**Fig 2:**
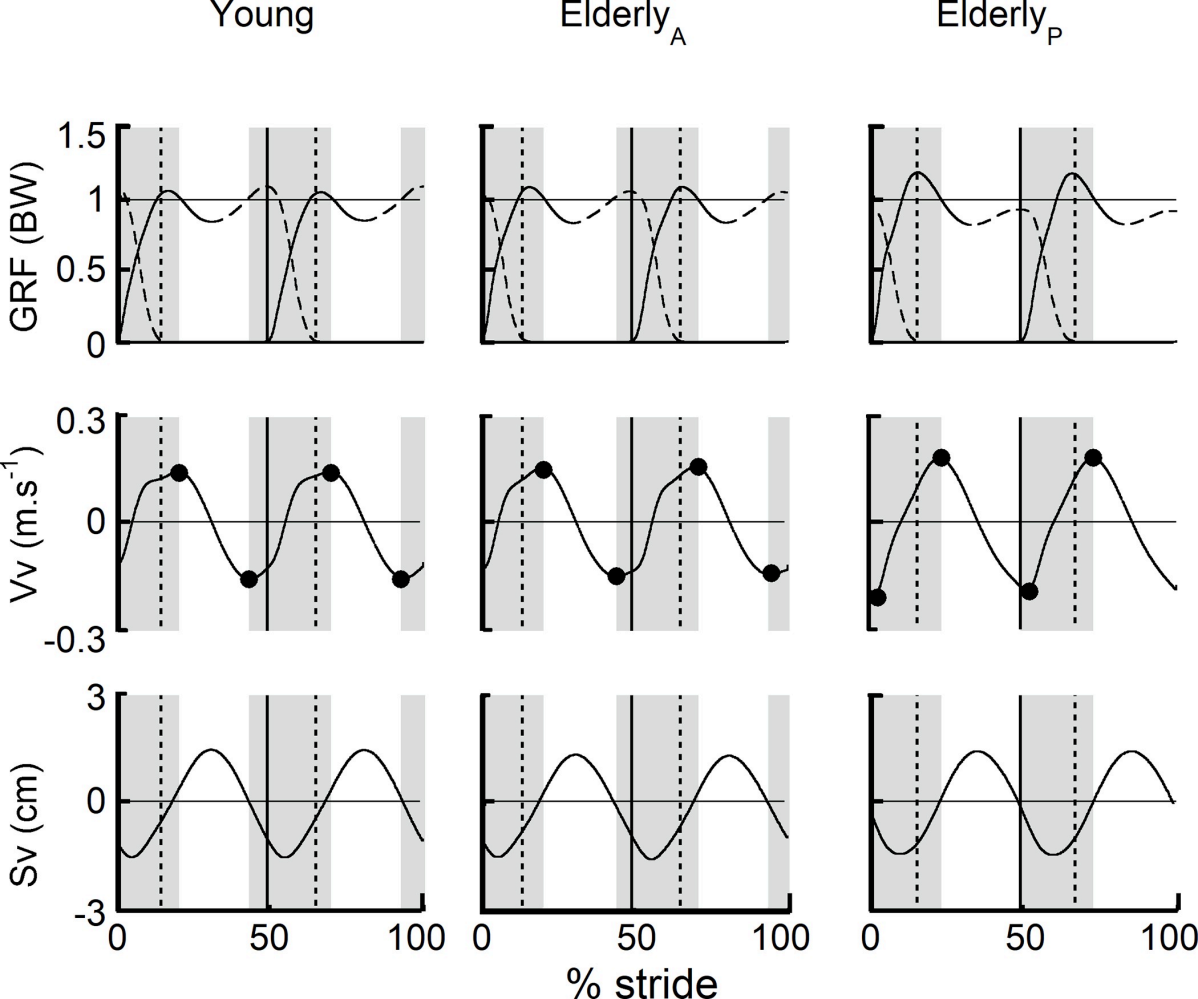
Kinetic curves for young and elderly adults with an active step-to-step transition (Elderly_A_) and with a passive step-to-step transition (Elderly_P_) during walking. Mean curves of the vertical component of the ground reaction force (GRF, expressed in body weight) acting upon each leg separately (continuous line: front leg; dotted line: back leg), of the vertical component of the velocity of the CoM (Vv, expressed in m.s^-1^) and its displacement (Sv, expressed in cm) during a stride for the three groups walking at 1.11 m.s^-1^. The vertical lines indicate the double contact phase (DC): the continuous lines correspond to the foot contact (FC) of the front leg and the dotted lines correspond to the toe-off (TO) of the back leg; the grey areas represent the step-to-step transition phase between the minimum of Vv before FC and the maximum of Vv after TO.

The Fig 2 presents the mean traces of the vertical ground reaction force (GRF) acting upon each leg separately, the vertical component of the velocity (Vv) and the vertical displacement of the CoM during a stride at 1.11 m.s^-1^ for young adults and elderly adults with an active transition or with a passive transition (Elderly_A_ and Elderly_P_, respectively). In young adults and Elderly_A_, the vertical push-off of the back leg becomes superior to one body weight value before FC. Consequently, the Vv_min_ and the upward redirection of the CoM occur before FC (Fig 2). In Elderly_P_, the vertical push-off of the back leg never overpasses one body weight value and, consequently, the transition is initiated by FC: the minimum of Vv and the upward redirection of the CoM occur during the double contact phase. In Elderly_P,_ the vertical impact on the front leg is increased and the GRF pattern of the front vs back foot is clearly more asymmetric compared to young adults and Elderly_A_ (Fig 2).

**Fig 3:**
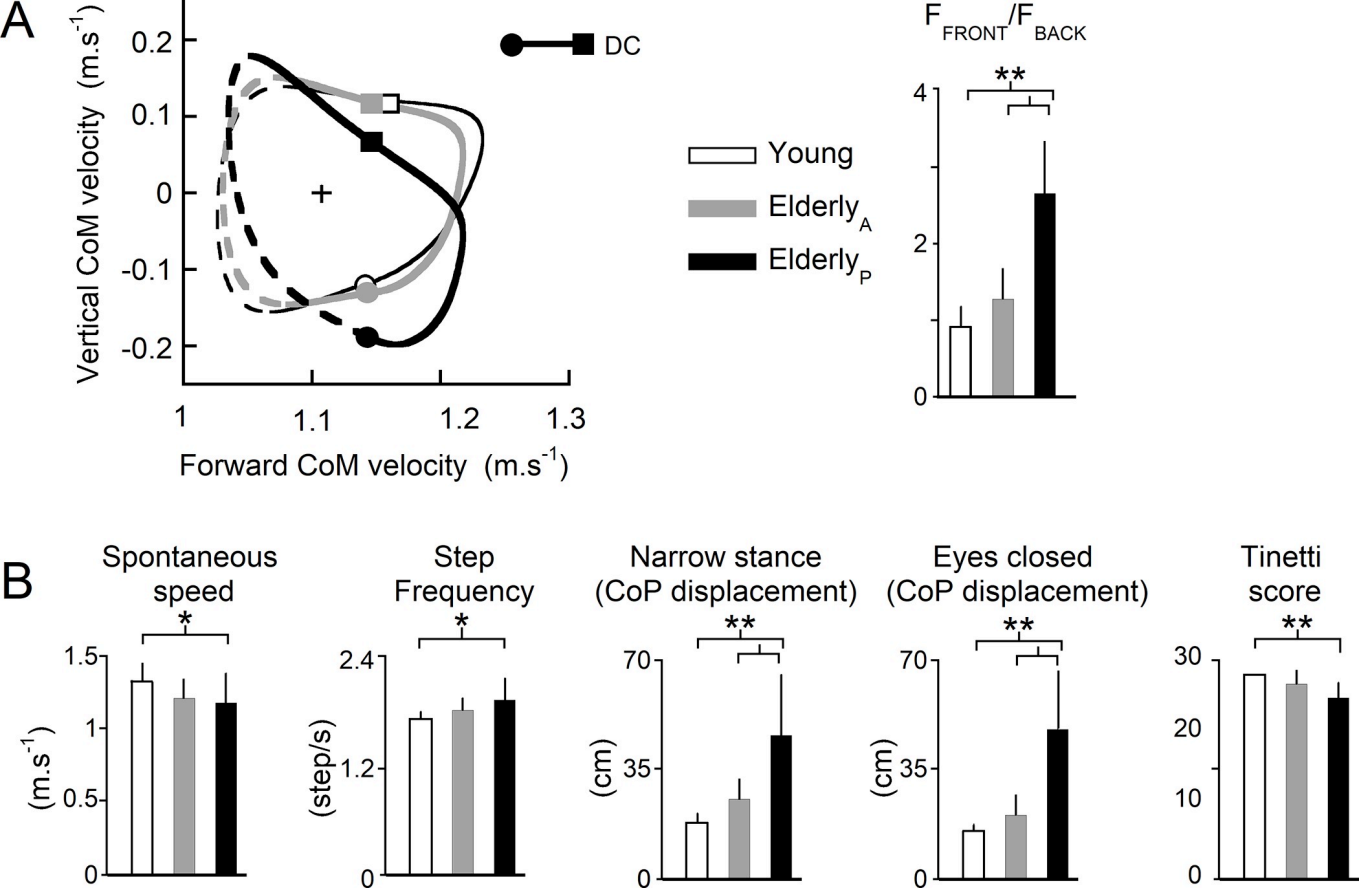
Results. (A) Left: mean plot of the vertical (Vv) and forward (Vf) components of the velocity of the CoM during a step at 1.11 m.s^-1^ for the 3 groups. Mean speed is represented by +. The step-to-step transition phase is illustrated by the continuous line. The double contact phase is delimited by the circle (front foot contact) and the square (back foot toe-off). Right: mean value (bar) +1 SD (tick) of the ratio F_FRONT_/F_BACK_ during the step-to-step transition. White: young adults; grey: elderly adults with active step-to-step transition; black: elderly adults with passive step-to-step transition. (*): p ≤ 0.05, (**): p ≤ 0.01. N = 1270 weight transfer (young: 351; Elderly_A_: 523 and Elderly_P_: 396; transfers from the right leg to the left: 628; from the left leg to the right: 640). (B) Mean values of the spontaneous speed (m.s^-1^), the step frequency (step/s), the displacement of the center of pressure (CoP, in cm) during standing with a narrow stance and with eyes closed, and the Tinetti score. Same data as Fig 3A for the step frequency and the spontaneous speed and N = 26 (young: 7; Elderly_A_: 14 and Elderly_P_: 5) for the displacement of the CoP during standing with narrow stance and eyes closed and for the Tinetti score.

The Fig 3 presents the differences between the three groups for the kinetic, speed-related and balance parameters. The left part of Fig 3A presents the mean plot of the vertical (Vv) and the forward component (Vf) of the CoM velocity during a step. Elderly_P_ present a distorted hodograph in comparison to young and Elderly_A_ highlighting a modification of the relationship between Vf and Vv throughout the step. Moreover, Elderly_P_ have a significant greater F_FRONT_/F_BACK_ ratio in comparison to the two other groups with the mean force of the front leg two and a half times greater than the mean force of the back leg (right part of Fig 3A, F_FRONT_/F_BACK_, group effect p<0.001).

### Balance and speed-related parameters

The Fig 3B illustrates the differences in speed-related parameters (step frequency and spontaneous speed), balance parameters (standing with eyes closed and standing with a narrow stance) and the Tinetti score between the 3 groups. The mean spontaneous speed is equal to 1.32±0.13, 1.20±0.13 and 1.17±0.20 m.s^-1^ for young, Elderly_A_ and Elderly_P_, respectively, with a significant difference between young and Elderly_P_ (Fig 3B, group effect: p<0.05). Similarly, Elderly_P_ have significant greater step frequency and significant smaller Tinetti score in comparison to young adults (Fig 3B, group effect p<0.05) but there is no significant difference with Elderly_A_. On the contrary for the two balance parameters, there is a significant difference between the two groups of elderly adults. Indeed, Elderly_P_ have greater displacement of the CoP during standing with eyes closed and standing with a narrow stance in comparison to the two other groups (Fig 3B, group effect p<0.01).

### Balance, speed-related and transition parameters relationships

The Table 1 shows the correlations between speed-related, balance and the transition parameters (F_FRONT_/F_BACK_ ratio and the time of occurrence of Vv_min_ relative to FC). There are strong correlations between the transition and the balance parameters, while there is no correlation between the speed-related parameters and the transition parameters or the balance parameters.

**Table 1:** Correlations between speed-related, balance and transition parameters.

		Speed-related		Balance		Transition
		Spontaneous speed	Step frequency	CoP eyes closed	CoP narrow stance	Vv_min_ relative to FC
Speed-related	Step frequency	-,418*				
Balance	CoP eyes closed	-.136	-.179			
	CoP narrow stance	-.076	.016	,753**		
Transition	Vv_min_ relative to FC	.313	-.340	-,584**	-,593**	
	F_FRONT_/F_BACK_	-.164	.163	,602**	,742**	-,834**

N = 26 (young: 7; Elderly_A_: 14 and Elderly_P_: 5).

(*): p ≤ 0.05

(**): p ≤ 0.01.

## Discussion

The aim of this study is to use the transition mode to identify two groups in the aged population. For this study, the participants were asked to walk at a controlled speed because the transition mode is speed-dependent. Indeed, young adults have a passive transition at slow speeds and an active transition at medium and high speeds [24]. Consequently, a comparison at uncontrolled speed does not allow determining if the modification of the walking pattern comes from a reduction of the speed or a modification of the balance control. The chosen speed of 1.11 m.s^-1^ corresponds to a speed where young adults have an active transition and is close to the spontaneous speed we measured in our elderly population.

The transition mode identifies two groups of elderly adults: some with an active transition (Elderly_A_) and others with a passive transition (Elderly_P_). Our results show in elderly adults an effect of the transition mode on the kinetic and balance parameters but not on speed-related parameters and the Tinetti score. Indeed, only the F_FRONT_/F_BACK_ ratio and the balance parameters (the displacement of the center of pressure during standing with a narrow stance and with eyes closed) are correlated with the transition mode.

In Elderly_A_, the F_FRONT_/F_BACK_ ratio shows an almost equal contribution of the back and the front legs to the transition and the shape of hodograph is similar in comparison to young adults: the relationship between Vf and Vv is not modified. In Elderly_P_, the F_FRONT_/F_BACK_ ratio shows a greater force developed by the front leg than by the back leg during the transition and the relationship between Vf and Vv is modified. These kinetic differences between Elderly_A_ and Elderly_P_ highlight two different strategies in the balance control to achieve the transfer of the body weight from one leg to the other.

The transition mode is determined by the vertical push-off: a push-off inferior to one body weight will result in a passive transition mode while a push-off superior to one body weight will result in an active transition mode [29]. As a matter of fact, the major determinants of the transition mode are the plantar flexors of the back leg since they are the greatest contributors to the vertical push-off [30]. A reduction of the plantar flexion in elderly adults is commonly observed in the literature [31] and is attributed to an underutilisation of the available muscular capacity [32] and/or a failure in the interaction between brain, interneurons, motoneurons, and sensory feedback [33]. This fail seems to emerge with age and one of the possible causes of passive transition could be a functional decline in the interactions between these different neurological systems. Indeed, the passive transition has already been associated with disrupted somatosensory inputs, progressive supranuclear palsy, dysfunction of descending basal ganglia outputs and atrophy of the mesencephalon [20, 34–36]. Interestingly, stimulation applied in the midbrain of Parkinson’s disease patients has been shown to improve the transition (i.e. the passive transition becomes active) and to decrease their balance disorders [19].

The static balance capacity seems modified in Elderly_P_; they exhibit a greater CoP excursion during standing with eyes closed and standing with narrow stance compared to Elderly_A_. These two balance parameters are associated with fall history and balance modification in elderly adults [12, 37, 38]. More precisely, these two tests reflect the ability of the postural control system to correctly perceive the environment through peripheral sensory system and integrate vestibular, visual and proprioceptive inputs at the central nervous system level [28]. Although the CoP was measured during 30 seconds while 90 seconds is recommended [28], the difference between Elderly_A_ and Elderly_P_ is significant. Standing with narrow stance increases the static balance challenge while standing with eyes closed prevents visual compensatory during balance control. The greater CoP excursions in Elderly_P_ and the strong relationship between these two parameters (Table 1) seem indicate a modification in the integration of sensory and/or vestibular inputs used during both standing balance tasks. The strong relationship between the balance parameters and the transition parameters (Vv_min_ relative to FC and the F_FRONT_/F_BACK_ ratio) could highlight that walking and static balances are controlled by common systems. On the other hand, the absence of relationship between the balance parameters and the speed-related parameters (step length and velocity) indicates that others systems control speed-related parameters of locomotion. The absence of modification for speed-related parameters with the stimulation in midbrain structure of Parkinson’s disease patients [19] corroborates our results.

According to Browne and Franz [39], balance is a general term describing the resilience to falling and the dynamic stability is a measure of balance specific to dynamic tasks such as walking. These authors quantified the dynamic stability via the maximum divergence Lyapunov exponent, calculated from the position and velocity of the sacrum. They have shown that young adults adopt propulsive force generation during the push-off that maximizes dynamic stability and suggested that a diminished push-off in elderly adults is in phase with poorer balance control. These results corroborate the relationship between the transition mode and the balance control we observe.

Finally, the use of transition mode for the prevention of first-fall presents several advantages. First, the transition mode is more sensitive to subtle modifications of the walking pattern before the first-fall than screening tools, as illustrated by the absence of significant difference between Elderly_A_ and Elderly_P_ for the Tinetti score. Second, the transition corresponds to a period of gait when most of falls occur [15, 16]. Third, the transition mode provides information on the balance control during walking and brings substantial complement to standing tests.

In conclusion, our results show that elderly adults could be sorted in two groups according to their active or passive step-to-step transition mode. The elderly adults with a passive transition present a disbalanced contribution of the front and back legs and a distorted hodograph that are correlated with modifications of postural balance controls. The emergence of a passive transition could be an early sign of functional decline of neurological systems controlling static and walking balances. The difference between the two groups suggests that the transition mode is a potential indicator of first-fall risk during walking. Future prospective studies should analyze its predictive value in the prevention of first-fall during walking.

## Supporting information

S1 Dataset Subject by subject data. (XLS)

## References

[1] Tromp AM, Pluijm SM, Smit JH, Deeg DJ, Bouter LM, Lips P. Fall-risk screening test: a prospective study on predictors for falls in community-dwelling elderly. Journal of clinical epidemiology. 2001;54(8):837–44. 11470394 10.1016/s0895-4356(01)00349-3

[2] Sterling DA, O'Connor JA, Bonadies J. Geriatric falls: injury severity is high and disproportionate to mechanism. The Journal of trauma. 2001;50(1):116–9. 10.1097/00005373-200101000-00021 11231681

[3] Gerdhem P, Ringsberg KA, Akesson K, Obrant KJ. Clinical history and biologic age predicted falls better than objective functional tests. Journal of clinical epidemiology. 2005;58(3):226–32. 10.1016/j.jclinepi.2004.06.013 15718110

[4] Deandrea S, Lucenteforte E, Bravi F, Foschi R, La Vecchia C, Negri E. Risk factors for falls in community-dwelling older people: a systematic review and meta-analysis. Epidemiology. 2010;21(5):658–68. 10.1097/EDE.0b013e3181e89905 20585256

[5] Konig N, Taylor WR, Armbrecht G, Dietzel R, Singh NB. Identification of functional parameters for the classification of older female fallers and prediction of 'first-time' fallers. Journal of the Royal Society, Interface. 2014;11(97):20140353 10.1098/rsif.2014.0353 24898021 PMC4208368

[6] Yardley L, Smith H. A prospective study of the relationship between feared consequences of falling and avoidance of activity in community-living older people. The Gerontologist. 2002;42(1):17–23. 10.1093/geront/42.1.17 11815695

[7] Gates S, Smith LA, Fisher JD, Lamb SE. Systematic review of accuracy of screening instruments for predicting fall risk among independently living older adults. Journal of rehabilitation research and development. 2008;45(8):1105–16. 19235113

[8] Tinetti ME. Performance-oriented assessment of mobility problems in elderly patients. Journal of the American Geriatrics Society. 1986;34(2):119–26. 10.1111/j.1532-5415.1986.tb05480.x 3944402

[9] Kopke S, Meyer G. The Tinetti test: Babylon in geriatric assessment. Zeitschrift fur Gerontologie und Geriatrie. 2006;39(4):288–91. 10.1007/s00391-006-0398-y 16900448

[10] Miodonska Z, Stepien P, Badura P, Choroba B, Kawa J, Derejczyk J, et al Inertial data-based gait metrics correspondence to Tinetti Test and Berg Balance Scale assessments. Biomedical Signal Processing and Control. 2018;44:38–47.

[11] Panella L, Lombardi R, Buizza A, Gandolfi R, Pizzagalli P. Towards objective quantification of the Tinetti test. Functional neurology. 2002;17(1):25–30. 12086109

[12] Melzer I, Benjuya N, Kaplanski J. Postural stability in the elderly: a comparison between fallers and non-fallers. Age and ageing. 2004;33(6):602–7. 10.1093/ageing/afh218 15501837

[13] Nutt JG, Marsden CD, Thompson PD. Human walking and higher-level gait disorders, particularly in the elderly. Neurology. 1993;43(2):268–79. 10.1212/wnl.43.2.268 8437689

[14] Lord S, Galna B, Rochester L. Moving forward on gait measurement: toward a more refined approach. Movement disorders: official journal of the Movement Disorder Society. 2013;28(11):1534–43. 24132841 10.1002/mds.25545

[15] Blake AJ, Morgan K, Bendall MJ, Dallosso H, Ebrahim SB, Arie TH, et al Falls by elderly people at home: prevalence and associated factors. Age and ageing. 1988;17(6):365–72. 10.1093/ageing/17.6.365 3266440

[16] Robinovitch SN, Feldman F, Yang Y, Schonnop R, Leung PM, Sarraf T, et al Video capture of the circumstances of falls in elderly people residing in long-term care: an observational study. Lancet. 2013;381(9860):47–54. 10.1016/S0140-6736(12)61263-X 23083889 PMC3540102

[17] Kuo AD, Donelan JM, Ruina A. Energetic consequences of walking like an inverted pendulum: step-to-step transitions. Exercise and sport sciences reviews. 2005;33(2):88–97. 15821430 10.1097/00003677-200504000-00006

[18] Franz JR, Kram R. Advanced age affects the individual leg mechanics of level, uphill, and downhill walking. Journal of biomechanics. 2013;46(3):535–40. 10.1016/j.jbiomech.2012.09.032 23122946 PMC3616147

[19] Chastan N, Westby GW, Yelnik J, Bardinet E, Do MC, Agid Y, et al Effects of nigral stimulation on locomotion and postural stability in patients with Parkinson's disease. Brain: a journal of neurology. 2009;132(Pt 1):172–84. 19001482 10.1093/brain/awn294

[20] Welter ML, Do MC, Chastan N, Torny F, Bloch F, du Montcel ST, et al Control of vertical components of gait during initiation of walking in normal adults and patients with progressive supranuclear palsy. Gait & posture. 2007;26(3):393–9. 17126017 10.1016/j.gaitpost.2006.10.005

[21] Chong RK, Chastan N, Welter ML, Do MC. Age-related changes in the center of mass velocity control during walking. Neuroscience letters. 2009;458(1):23–7. 10.1016/j.neulet.2009.04.022 19442871

[22] Adamczyk PG, Kuo AD. Redirection of center-of-mass velocity during the step-to-step transition of human walking. The Journal of experimental biology. 2009;212(Pt 16):2668–78. 10.1242/jeb.027581 19648412 PMC2726857

[23] Breniere Y, Bril B. Development of postural control of gravity forces in children during the first 5 years of walking. Experimental brain research. 1998;121(3):255–62. 10.1007/s002210050458 9746131

[24] Meurisse GM, Bastien GJ, Schepens B. Effect of age and speed on the step-to-step transition phase during walking. Journal of biomechanics. 2019;83:253–9. 10.1016/j.jbiomech.2018.12.001 30554814

[25] Chastan N, Debono B, Maltete D, Weber J. Discordance between measured postural instability and absence of clinical symptoms in Parkinson's disease patients in the early stages of the disease. Movement disorders: official journal of the Movement Disorder Society. 2008;23(3):366–72. 18044726 10.1002/mds.21840

[26] Meurisse GM, Dierick F, Schepens B, Bastien GJ. Determination of the vertical ground reaction forces acting upon individual limbs during healthy and clinical gait. Gait & posture. 2016;43:245–50. 26549482 10.1016/j.gaitpost.2015.10.005

[27] Cavagna GA. Force platforms as ergometers. J Appl Physiol. 1975;39(1):174–9. 10.1152/jappl.1975.39.1.174 1150585

[28] Ruhe A, Fejer R, Walker B. The test-retest reliability of centre of pressure measures in bipedal static task conditions—a systematic review of the literature. Gait & posture. 2010;32(4):436–45. 20947353 10.1016/j.gaitpost.2010.09.012

[29] Honeine JL, Schieppati M, Gagey O, Do MC. The functional role of the triceps surae muscle during human locomotion. PloS one. 2013;8(1):e52943 10.1371/journal.pone.0052943 23341916 PMC3547017

[30] Schloemer SA, Thompson JA, Silder A, Thelen DG, Siston RA. Age-Related Differences in Gait Kinematics, Kinetics, and Muscle Function: A Principal Component Analysis. Annals of biomedical engineering. 2017;45(3):695–710. 10.1007/s10439-016-1713-4 27573696

[31] Boyer KA, Johnson RT, Banks JJ, Jewell C, Hafer JF. Systematic review and meta-analysis of gait mechanics in young and older adults. Experimental gerontology. 2017;95:63–70. 10.1016/j.exger.2017.05.005 28499954

[32] Franz JR. The Age-Associated Reduction in Propulsive Power Generation in Walking. Exercise and sport sciences reviews. 2016;44(4):129–36. 10.1249/JES.0000000000000086 27433977 PMC9382873

[33] Honeine JL, Schieppati M, Gagey O, Do MC. By counteracting gravity, triceps surae sets both kinematics and kinetics of gait. Physiological reports. 2014;2(2):e00229 10.1002/phy2.229 24744898 PMC3966244

[34] Chastan N, Do MC, Bonneville F, Torny F, Bloch F, Westby GW, et al Gait and balance disorders in Parkinson's disease: impaired active braking of the fall of centre of gravity. Movement disorders: official journal of the Movement Disorder Society. 2009;24(2):188–95. 18973252 10.1002/mds.22269

[35] Chastan N, Westby GW, du Montcel ST, Do MC, Chong RK, Agid Y, et al Influence of sensory inputs and motor demands on the control of the centre of mass velocity during gait initiation in humans. Neuroscience letters. 2010;469(3):400–4. 10.1016/j.neulet.2009.12.038 20026383

[36] Demain A, Westby GW, Fernandez-Vidal S, Karachi C, Bonneville F, Do MC, et al High-level gait and balance disorders in the elderly: a midbrain disease? Journal of neurology. 2014;261(1):196–206. 10.1007/s00415-013-7174-x 24202784 PMC3895186

[37] Maki BE, Holliday PJ, Topper AK. A prospective study of postural balance and risk of falling in an ambulatory and independent elderly population. Journal of gerontology. 1994;49(2):M72–84. 8126355 10.1093/geronj/49.2.m72

[38] Edginton Bigelow K, Berme N. Development of a Protocol for Improving the Clinical Utility of Posturography as a Fall-Risk Screening Tool. The Journals of Gerontology: Series A. 2010;66A(2):228–33. 10.1093/gerona/glq202 21127191

[39] Browne MG, Franz JR. Does dynamic stability govern propulsive force generation in human walking? Royal Society open science. 2017;4(11):171673 10.1098/rsos.171673 29291129 PMC5717707

