# Correction: The step-to-step transition mode: A potential indicator of first-fall risk in elderly adults?

**DOI:** 10.1371/journal.pone.0223321

**Published:** 2019-09-26

**Authors:** The PLOS ONE Staff

The Abstract section is omitted from the article. The publisher apologizes for the error. Please see the complete Abstract section here.

## Abstract

Gait analysis is a powerful tool to identify markers of functional decline throughout ageing. A failed weight transfer from one leg to the other during walking, i.e. during the transition between steps, is one of the main causes of falls in elderly adults. Two modes of transition are observed: an active mode when the transition begins before the double contact phase and a passive mode when the transition begins during the double contact phase. The aim of this study is to identify groups in the aged population based on the step-to-step transition mode. Fifteen young and thirty-six elderly adults walked on a force-measuring treadmill at 1.11 m.s^-1^. Seven (out of fifteen) young and nineteen (out of thirty-six) elderly adults also performed static balance tasks to evaluate their postural stability during standing. Results show that elderly adults could be sorted into the two groups. The elderly adults with a passive transition present a disbalanced contribution of the front and back legs during the transition and a distorted hodograph representing the vertical and forward components of the velocity of the body plotted against each other. These kinetic modifications are correlated to greater center of pressure excursions during the standing tasks suggesting that walking and static balance are controlled by common systems. This leads us to suggest the transition mode as an attractive indicator of first-fall risk during walking. The emergence of a passive transition could be an early sign of functional decline of neurological systems controlling static and dynamic balances. Future prospective studies should analyze its predictive value in the prevention of first-fall during walking.
